# The Incidence of Acute Gastrointestinal Illness in Canada, Foodbook Survey 2014-2015

**DOI:** 10.1155/2017/5956148

**Published:** 2017-12-19

**Authors:** M. Kate Thomas, Regan Murray, Andrea Nesbitt, Frank Pollari

**Affiliations:** Centre for Foodborne, Environmental and Zoonotic Infectious Diseases, Public Health Agency of Canada, Guelph, ON, Canada

## Abstract

Acute gastrointestinal illness (AGI) is an important public health issue, with many pathogen sources and modes of transmission. A one-year telephone survey was conducted in Canada (2014-2015) to estimate the incidence of self-reported AGI in the previous 28 days and to describe health care seeking behaviour, using a symptom-based case definition. Excluding cases with respiratory symptoms, it is estimated that there are 0.57 self-reported AGI episodes per person-year, almost 19.5 million episodes in Canada each year. The proportion of cases seeking medical care was nearly 9%, of which 17% reported being requested to submit a sample for laboratory testing, and 49% of those requested complied and provided a sample. Results can be used to inform burden of illness and source attribution studies and indicate that AGI continues to be an important public health issue in Canada.

## 1. Introduction

Acute gastrointestinal illness (AGI) is an important public health issue, with substantial economic and human health impact [1–4]. Numerous countries in Europe, North and South America, Asia, and Australia and New Zealand have conducted population-based studies to estimate the incidence and burden of AGI in the community [5–21]. In Canada, the National Studies on Acute Gastrointestinal Illness (NSAGI) initiative was initiated in 1999 and developed population estimates in select regions of Canada [22–24]. The NSAGI studies have been used to inform Canadian burden of enteric illness studies [25–27].

In 2014-15, a population-based telephone survey, the Foodbook study, was conducted nationally to describe Canadians' exposure to foods, animals, and water that may serve as sources for enteric illness pathogens and included questions related to AGI symptoms and care seeking behaviours [28]. The objective of this paper is to describe the prevalence, distribution, and symptoms of AGI and the health care seeking behaviours of individuals with AGI, across Canada for 2014-15 based on the Foodbook study.

## 2. Materials and Methods

### 2.1. Study Design and Data Collection

The Foodbook study was conducted in Canada's 10 provinces and 3 territories over a one-year period (April 2014-April 2015) using a population-based telephone survey and included questions on AGI symptoms and related health care seeking behaviours. Households were randomly selected from a sampling frame of telephone numbers that consisted of land lines (70% listed and 10% random digit-dialing) and cell phones (20%). The survey was designed with a target total sample size of 11,016 surveys collected evenly over a 12-month period across four age groups (0–9, 10–19, 20–64, and 65+ years) and all 13 Canadian provinces and territories. In order to improve completion rates for younger age groups, when households contained children less than 18 years old, 50% of surveys were conducted with the child who would have the next birthday and 50% were conducted with the adult who would have the next birthday. If there were no children in the household, the survey was conducted with the adult who would have the next birthday. Complete details on participant selection and questionnaire administration can be found in the Foodbook Report [28]. The surveys were conducted by an independent research company contracted by the Public Health Agency of Canada (the Agency). Individuals were excluded if they could not speak the supported languages (English, French, Inuktitut, and on-demand verbal translation for other languages), if they did not have a listed land line or cellular telephone number or travelled outside their province or territory of residence during the seven days prior to interview. The Foodbook study was reviewed and approved by Health Canada and the Agency's Research Ethics Board (REB 2013-0025) as well as the Newfoundland and Labrador Health Research Ethics Authority to meet a unique provincial legal requirement (HREB 13.238).

Weighted selection of survey participants to reflect the Canadian population was assigned using the following method. The forward sortation area (FSA, or first three digits of the postal code) collected for each respondent was converted to the most likely census metropolitan area (CMA). Using 2011 Census data, the CMA indicator along with age group, household type, province or territory, the number of people in the household, the number of land lines and cell phones in the household, and gender were used to calculate the individual-level survey expansion weight. To create the final weighting variable, a poststratification step used iterative proportional ranking with available control tables. The population reference year was 2011, representing a population of 33,400,000 [29].

The survey questions relating to AGI were developed to be consistent with the NSAGI studies previously conducted in Canada [22–24]. Respondents were asked if they had experienced any vomiting or diarrhea in the 28 days prior to the interview. The module included questions about symptoms, their frequency and duration, existing chronic conditions and medication use, respiratory symptoms, and care seeking and stool submission behaviours. The survey was pilot-tested over a two-week period before full survey implementation.

### 2.2. Case Definitions

Illnesses reported to have started prior to the 28 days of the interview were excluded (3.6% weighted). Respondents who identified more than one episode of AGI during the 28 days prior to the interview were asked to respond only for their most recent episode. Respondents who did not report symptoms of AGI, as well as those identified as having self-reported that their diarrhea or vomiting was due to pregnancy, medical treatment (e.g., chemotherapy), or medical conditions (e.g., Crohn's disease, colitis, irritable bowel syndrome, and alcoholism), were included in the noncase category.

Two case definitions of AGI were assessed: (a) a person reporting three or more loose stools in 24 hours or any vomiting in the past 28 days, according to the international AGI definition [24] and (b) a person reporting three or more loose stools in 24 hours or any vomiting in the past 28 days without concurrent respiratory symptoms (cough or sore throat) [25]. Removing cases with respiratory symptoms creates a more specific definition attempting to exclude respiratory infections that may cause gastrointestinal symptoms such as vomiting or diarrhea [30].

### 2.3. Missing Data and Resolution

Starting in November 2014 to the end of the survey time period (April 2015), due to an error in the Computer Assisted Telephone Interviewing (CATI) survey tool, 225 respondents who indicated AGI symptoms were not asked all the relevant survey questions. These participants responded “yes” to having any symptoms of vomiting or diarrhea but “no” to vomiting symptoms specifically; therefore, it is assumed that they only had diarrhea symptoms. Questions missed pertained to the subsection specific to diarrhea and included duration of diarrhea symptoms, number of stools, and related chronic conditions or medication use.

An adjustment for missing data was made by weighting completed interviews for respondents that reported symptoms of diarrhea only (April 2014 to October 2014; weighted *n* = 988,114.51) to account for the missing data (November 2014 to April 2015; weighted *n* = 855,500.56) by province (and respiratory symptoms for the more specific case definition), therefore increasing the weight assigned to the completed interviews (Appendix A). An assumption was made that there would be similar chronic disease and duration of symptoms between the diarrhea-only cases from April to October and November to April.

## 3. Analysis

Data analysis was performed in Stata 13.0 (StataCorp., Texas Station, TX) using the survey weight, and only weighted results are reported. Categorical variables were described using weighted percentages and the relative 95% confidence interval (CI). Individuals responding “don't know” or “refused” to any question were excluded from the analysis of that question. Mean and median values were used to describe continuous variables.

For incidence rate calculations, respondents identifying multiple episodes were counted as a single episode. The primary outcome measure of monthly prevalence was defined as the number of respondents reporting AGI in the previous 28 days divided by the total number of respondents. Annual incidence rate and incidence proportion calculations were performed using formulas found in Appendix B [31].

The null hypothesis of no overall association between the prevalence of disease and province was tested using the Wald χ^2^ test, with a *p* value cutoff of 0.05. The difference between the proportion of cases (i.e., the prevalence of AGI) in a specific province and the proportion of cases in all other provinces combined was tested using the χ^2^ test.

## 4. Results

The survey response rate was 19.9%, and a total of 10,798 residents responded to the survey (Table 1). There were 975 respondents (weighted *n* = 2,803,946) that indicated symptoms of diarrhea or vomiting in the past 28 days, reflecting a monthly prevalence of 8.5%. Of these respondents, 12% (weighted) reported that their diarrhea or vomiting in the past 4 weeks was caused by a medical condition, medication, or pregnancy and were counted in the noncase group. Using the international AGI case definition of three or more loose stools in 24 hours and any vomiting [24], the overall monthly prevalence was 5.7% (95% CI 4.6–7.2, weighted *n* = 1,887,588) corresponding to an annual incidence rate of 0.77 episodes/person-year (95% CI 0.61–0.97) and 26.3 million episodes of AGI per year in Canada (Table 2). After removal of cases with concurrent respiratory symptoms (25%), the monthly prevalence was 4.3% (95% CI 3.1–5.8, weighted *n* = 1,407,698), with an annual incidence rate of 0.57 episodes/person-year (95% CI 0.41–0.78). This reflects 19.4 million episodes of AGI per year in Canada.

**Figure 1:**
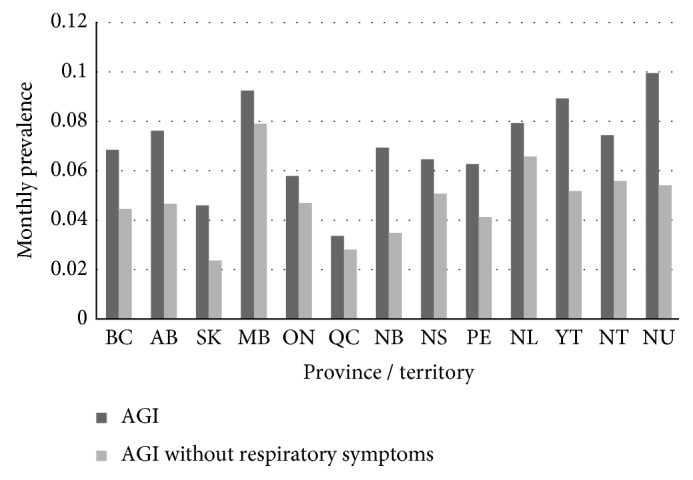
Monthly prevalence of self-reported AGI by province/territory, 2014-2015.

Estimates of monthly prevalence and incidence of AGI nationally and by province for both AGI case definitions are presented in Table 2. There were some regional differences identified: the monthly prevalence for the province of Quebec was significantly lower compared to the rest of the provinces/territories combined (*p* < 0.01) when assessing the international AGI case definition, and the monthly prevalence for the province of Saskatchewan was significantly lower compared to the rest of the provinces/territories combined (*p*=0.02) when assessing the more specific case definition of three or more loose stools in 24 hours and any vomiting without concurrent respiratory symptoms. A higher monthly prevalence was identified in Manitoba, Newfoundland, and the Territories; however, these differences were not statistically significant. The Territories and New Brunswick showed the greatest difference between prevalence of AGI when comparing AGI case definitions with and without concurrent respiratory symptoms (Figure 1).

**Figure 2:**
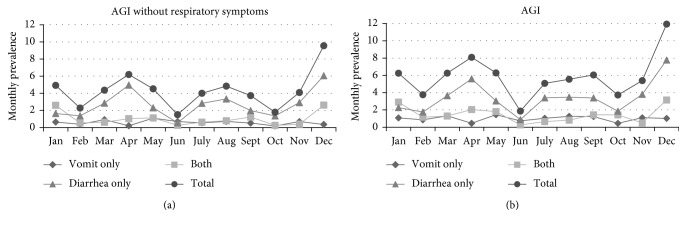
(a) and (b) Monthly prevalence of self-reported AGI by study month and symptoms, 2014-2015.

When considering predisposing factors, 5.6% of AGI cases took prescription antibiotics in the previous 28 days and 5.0% of AGI cases with no respiratory symptoms in the previous 28 days. There was no clear seasonal pattern: lower monthly prevalence in February, June, and October and higher monthly prevalence in December and April (Figure 2).

When assessing the most specific AGI case definition, 61.1% of respondents reported experiencing diarrhea symptoms only, while 24.3% reported both vomiting and diarrhea and 14.6% reported vomiting only (Table 3). Of the cases who experienced diarrhea, 9.9% reported bloody diarrhea (95% CI 3.4–25.8). Duration of symptoms was longest for those who experienced both vomiting and diarrhea compared to those experiencing only one symptom (Table 4). Cases of the more specific AGI definition reported a mean of 4.43 episodes of diarrhea and 3.97 episodes of vomiting in a 24-hour period.

When using the more specific AGI case definition to assess care seeking behaviour, overall, 8.8% (95% CI 4.9–15.1) of cases visited a physician (Table 5). Of these, 17.1% (95% CI 7.5–34.5) were requested to submit a stool sample by a physician, and 49.0% (95% CI 17.6–81.2) of these submitted a stool sample. Hospitalizations were reported by 0.68% of cases with a mean hospital stay of 3.38 days (median 2). Of all cases, 0.11% reported taking antibiotics to treat their illness.

## 5. Discussion

This is the first nationwide survey conducted in Canada to describe the magnitude and distribution of AGI in the general population. Based on the more specific definition of AGI, excluding cases with respiratory symptoms, it is estimated that there are 0.57 (95% CI 0.41–0.78) self-reported AGI episodes per person-year or almost 19.5 million episodes of AGI in Canada each year. This estimate is lower than the rate of 0.63 (95% CI 0.57–0.69) episodes per person-year that was estimated based on the combined previous NSAGI studies and used in the Canadian estimates of foodborne illnesses [25]; however, the 95% confidence intervals of the current and previous estimates overlap indicating a lack of statistical difference.

This lower estimated incidence in the current study year (2014/2015) compared to the previous NSAGI rates (2002–2006) may be related to the different approach in survey design, specifically the use of a weighted sampling technique. Other differences include the exclusion of respondents who travelled outside their province or territory of residence during the seven days prior to the interview in the current study that may have been experiencing symptoms. As well, a true lower incidence may be explained by epidemiological trends including variability in norovirus trends from year to year, the impact of rotavirus vaccine on illness associated with rotavirus [32, 33], or possibly other public health interventions.

The annual rate of AGI when using the specific definition and excluding respiratory symptoms is comparable to estimates from the United States (US) (individual population studies 0.49, 0.54, and 0.73 and overall 0.60 episodes per person-year) [34] and lower than Italy (0.76) [14]. When comparing the international AGI case definition, the Canadian annual incidence rate (0.77 episodes per person-year) is lower than Germany [12], Denmark [35], Italy [14], Chile [6], Australia, and the US [24], which ranged from 0.83 to 1.4 episodes per person-year, but is higher than Ireland (0.64) and Malta (0.37) [24]. The proportion of respondents excluded due to chronic conditions, medication use, or pregnancy as the cause of their symptoms in the present study (12%) was lower than that in the previous NSAGI studies (16–19%) [22, 23, 36].

Comparison of provincial/territorial results for Ontario, Quebec, and Nunavut using the international AGI definition showed that the estimates were lower than previous provincial/territorial illness estimates using the same definition [36–38]. The large variation in incidence between provinces/territories, though not statistically significant for many of them, does speak to the apparent regional differences of AGI incidence in Canada and the importance of capturing national information that reflects all provinces and territories. Furthermore, having provincial and territorial specific estimates enables individual jurisdictions to assess their AGI burden more specifically. This could be used to generate regional estimates of enteric illness and specific transmission routes (e.g., foodborne illness estimates for a specific province or territory) that could be used to inform public health activities (e.g., education and prevention campaigns).

The proportion of cases with respiratory symptoms (25%) is at the low end of the range reported by other countries reporting between 19% and 48% of cases experiencing concurrent respiratory symptoms [13, 14, 24, 35]. Using the more specific case definition creates a more conservative estimate, attempting to account for cases whose AGI symptoms may be caused by respiratory infections [14, 30, 34].

Diarrhea only was the predominant symptom profile of cases (55.9% and 61.1%, resp., for the two case definitions); this result falls between other studies, reporting a higher proportion in Germany (78%) and Denmark (64%) and lower proportions in Sweden, Italy, and Chile (30–40%) [6, 12, 14, 35, 39]. The proportion of diarrheal cases with bloody diarrhea (9.9%) was higher than that in other countries (3-4%) [5, 12, 13, 35]. This may be due to the small number of cases reporting bloody diarrhea and a large assigned weight due to study design.

The proportion of persons with AGI varied somewhat by season, which is similar to higher rates of AGI in winter months as what has been reported by previous Canadian studies [22, 40] as well as internationally in the US, Denmark, Italy, Sweden, and Germany [5, 12, 14, 35, 39]. This pattern is likely driven by viruses circulating in the winter months, particularly norovirus which is the most common cause of AGI in Canada [41]. The higher monthly prevalence observed in April may be an artefact due to the lower number of survey respondents in April (4% of the survey) compared to other months (approximately 8% each).

The percentage of cases who reported seeing a physician was low with only 8.8% seeking care, and 17.1% of these were requested to submit a stool sample. These values are weighted and are lower than those in the previous NSAGI studies (11–23% and 26–54%) [22, 23, 36]. The previous NSAGI studies were not age adjusted; therefore, the more frequent care seeking among the elderly may contribute to the higher overall results in the previous studies. The exclusion of individuals who travelled in the past seven days in the Foodbook study may also influence the lower results. Recent travel is associated with seeking medical care and having a sample requested [42, 43]; thus, these individuals who travelled may have been more likely to seek care and be requested to submit a stool sample. Lower care seeking rates would influence pathogen-specific estimates as it indicates greater underdiagnosis of cases. This should be considered among future burden of illness activities and how survey respondent weighting may influence this phenomenon.

Possible limitations of this study include the retrospective study design as it may be subject to recall bias. Retrospective studies in the UK (IID2) gave higher estimates of disease burden than prospective studies [19]. However, retrospective studies with longer recall periods gave lower estimated rates than studies with shorter recall periods [44]. Extrapolation from a reported seven-day prevalence was almost twice the rate of illness estimated when extrapolating from the month recall period [6, 35, 45].

The study response rate of 20% is lower than that in previous NSAGI studies [22, 40] and may be a source of bias if those who did not respond had different symptom profiles compared to those who participated in the study. Furthermore, misclassification of cases may have occurred due to excluding cases with chronic conditions or respiratory symptoms that might have been true infectious AGI cases.

The missing data for diarrheal cases from November to April due to the survey interview error were adjusted for based on known diarrheal cases captured from April to October; this however may not have accurately reflected the symptoms and behaviours of the missing cases and may have impacted the results. An assumption was made that there would be similar patterns (e.g., chronic disease and medical causes of symptoms, duration of symptoms, and care seeking behaviours) between the diarrhea-only cases from April to October and November to April. However, there may have been seasonal differences due to different pathogens circulating (e.g., norovirus in the winter or bacterial pathogens in the summer) or behavioural patterns (e.g., international winter travel or domestic summer recreational water exposure). From previous NSAGI studies, the monthly prevalence of AGI fluctuates seasonally with peaks seen in winter/early spring and again in summer [22–24]. Symptom-specific monthly variations were observed in Ontario where diarrhea only was the predominant symptom for most months, followed by both vomiting and diarrhea combined; however, the statistical significance of these variations was not reported, and the general relationship between symptom profiles does not vary much across seasons [23]. The adjustment was made based on province/territory only as there were insufficient data to allow for age-gender-province/territory-based adjustments. Differences due to age and gender would be inherently incorporated into the province-based adjustment. However, age- and gender-specific results could not be described due to this adjustment approach.

Generating an estimate of the total amount of AGI in Canada provides the foundation for pathogen- and transmission route-specific burden of illness estimates. The lower incidence of AGI reported here will inform future activities to refine estimates of food and waterborne illness in Canada. Additionally, provincial- and territorial-specific estimates will enable individual jurisdictions to assess their AGI burden and generate region-specific public health plans that could include, for example, focused education campaigns, public health policies, or resource allocations toward prevention of AGI strategies.

## Figures and Tables

**Table 1:** Demographic characteristics of survey participants in the Foodbook survey, Canada 2014-2015.

	*n*	Weighted (*n*)	Weighted (%)	Canadian population
Total	10,798	33,131,795	—	—
Gender				
Female	6,027	16,271,015	49.1	50.4
Male	4,771	16,860,780	50.9	49.6
Age group (years)				
0 to 9	2,460	3,674,744	11.1	10.8
10 to 19	2,345	4,139,321	12.5	12.0
20 to 64	3,080	20,428,351	61.7	62.6
65+	2,913	4,889,378	14.8	14.5
Province/territory				
British Columbia (BC)	1,258	4,409,227	13.3	13.1
Alberta (AB)	1,248	3,622,607	10.9	10.9
Saskatchewan (SK)	833	1,029,020	3.1	3.1
Manitoba (MB)	825	1,180,625	3.6	3.6
Ontario (ON)	1,645	12,769,166	38.5	38.4
Quebec (QC)	1,661	7,853,684	23.7	23.6
New Brunswick (NB)	609	752,454	2.3	2.2
Nova Scotia (NS)	627	904,078	2.7	2.8
Prince Edward Island (PE)	440	140,308	0.4	0.4
Newfoundland (NL)	434	517,646	1.6	1.5
Yukon (YT)	396	33,828	0.1	0.1
Northwest Territories (NT)	455	41,785	0.1	0.1
Nunavut (NU)	367	32,339	0.1	0.1

**Table 2:** Monthly prevalence (95% CI) and annual incidence rate (95% CI) of self-reported acute gastrointestinal illness (AGI) in Canada by province, 2014-2015.

	AGI				AGI without respiratory symptoms			
	Adjusted monthly prevalence (%)	95% CI	Annual incidence per person-year	95% CI	Adjusted monthly prevalence (%)	95% CI	Annual incidence per person-year	95% CI
Overall province	5.7	4.6–7.2	0.77	0.61–0.97	4.3	3.1–5.8	0.57	0.41–0.78
BC	6.8	4.3–10.8	0.92	0.57–1.49	4.5	2.5–7.8	0.59	0.33–1.06
AB	7.6	4.9–11.6	1.03	0.65–1.61	4.7	3.0–7.3	0.62	0.40–0.99
SK	4.6	2.8–7.3	0.61	0.37–0.99	**2.4**	**1.6–3.5**	**0.31**	**0.21–0.46**
MB	9.2	4.8–17.0	1.26	0.64–2.42	7.9	3.7–16.3	1.07	0.49–2.31
ON	5.8	3.6–9.3	0.78	0.48–1.27	4.7	2.5–8.7	0.63	0.33–1.19
QC	**3.4**	**2.1–5.4**	**0.45**	**0.28–0.72**	2.8	1.6–4.9	0.37	0.21–0.65
NB	6.9	4.4–10.7	0.94	0.59–1.47	3.5	1.8–6.5	0.46	0.24–0.88
NS	6.5	4.4–9.3	0.87	0.59–1.27	5.1	3.5–7.8	0.68	0.46–1.06
PE	6.3	3.2–11.9	0.84	0.42–1.65	4.1	2.9–8.8	0.55	0.38–1.20
NL	7.9	4.6–13.3	1.08	0.61–1.86	6.6	3.5–12.1	0.89	0.46–1.68
YT	8.9	4.4–17.3	1.22	0.59–2.47	5.2	2.9–9.2	0.69	0.38–1.26
NT	7.4	4.3–12.6	1.01	0.57–1.75	5.6	2.9–10.6	0.75	0.38–1.46
NU	9.9	5.3–17.9	1.36	0.71–2.56	5.4	2.2–12.6	0.72	0.29–1.75

Bold indicates monthly prevalence per category level significantly different from all other categories combined.

**Table 3:** Symptoms experienced by respondents reporting acute gastrointestinal illness in Canada, 2014-2015.

	AGI		AGI without respiratory symptoms	
Symptoms	Weighted (%)	95% CI	Weighted (%)	95% CI
% diarrhea and vomiting	25.6	18.1–34.9	24.3	9.6–21.8
% diarrhea only	55.9	44.9–66.2	61.1	47.3–73.3
% vomiting only	18.5	13.5–24.9	14.6	9.6–21.8
% with ongoing symptoms at time of interview	5.8	3.3–10.1	6.5	3.2–12.7
% diarrhea cases with bloody diarrhea	8.0	2.9–20.2	9.9	3.4–25.7

**Table 4:** Duration and frequency of symptoms of acute gastrointestinal illness in Canada, 2014-2015.

	AGI			AGI without respiratory symptoms		
	Mean	95% CI	Median	Mean	95% CI	Median
Both vomiting and diarrhea (days)	2.71	2.04–3.38	2	2.60	1.68–3.51	2
Duration of vomiting only (days)	1.19	1.08–1.29	1	1.18	1.03–1.33	1
Duration of diarrhea only (days)	2.59	1.22–3.96	1	1.60	1.26–1.94	1
Number of stools in 24 hours	4.48	3.97–4.99	4	4.43	3.87–4.99	4
Number of vomiting episodes in 24 hours	3.56	2.88–4.24	3	3.97	3.07–4.87	3

**Table 5:** Health care seeking, stool submission, and medication use of acute gastrointestinal illness cases in Canada, 2014-2015.

	AGI		AGI without respiratory symptoms	
		95% CI		95% CI
% of cases who saw physician	14.5	9.0–22.4	8.8	4.9–15.1
% of cases who sought care that were requested to submit a stool sample	12.7	6.2–24.4	17.1	7.5–34.5
% of cases submitted sample after physician request to submit	68.9	36.5–89.5	49.0	17.6–81.2
% of cases admitted to a hospital	1.1	0.45–2.7	0.68	0.28–1.7
Mean duration of hospital stay in days (median)	2.66 (1)		3.38 (2)	
% of cases reported antibiotic use prior to illness	5.6	2.9–10.3	5.0	2.0–11.9
% of all cases reported taking antibiotics to treat illness	0.55	0.24–1.3	0.11	0.01–0.79

**Table 6:** Input values used to generate province-based adjustment for missing interviews for AGI and AGI without respiratory symptoms.

	Adjustment 1				Adjustment 2			
	Complete diarrhea-only interviews (*A*) (*n,* weighted *n*)	Missing diarrhea-only interviews (*B*) (*n,* weighted *n*)	Total (*A* + *B*) (*n,* weighted *n*)	AGI multiplier (*C*)	Complete diarrhea only without respiratory symptom interviews (*X*) (*n,* weighted *n*)	Missing diarrhea only without respiratory symptom interviews (*Y*) (*n,* weighted *n*)	Total (*X* + *Y*) (*n,* weighted *n*)	AGI with no respiratory symptom multiplier (*Z*)
BC	39	30	69		30	19	49	
	172,666.37	85,951.20	258,617.57	1.50	159,460.83	44,916.74	204,377.57	1.28
AB	39	28	67		29	17	46	
	111,307.51	108,067.96	219,375.47	1.97	69,543.31	90,277.38	159,820.68	2.30
SK	26	21	47		20	9	29	
	25,989.24	19,463.92	45,453.15	1.75	16,146.28	6,643.13	22,789.42	1.41
MB	32	22	54		27	16	43	
	81,234.90	30,765.86	112,000.76	1.38	66,856.83	26,948.25	93,805.08	1.40
ON	46	31	77		31	22	53	
	439,337.45	375,169.93	814,507.38	1.85	258,982.59	287,359.19	546,341.78	2.11
QC	18	20	38		14.00	16	30	
	88,899.02	184,942.48	273,841.50	3.08	77,840.24	170,443.22	248,283.46	3.19
NB	18	15	33		11	6	17	
	19,749.42	14,894.60	34,644.03	1.75	9,978.81	5,097.38	15,076.19	1.51
NS	20	16	36		17	11	28	
	18,592.10	28,874.64	47,466.75	2.55	16,415.11	24,850.62	41,265.73	2.51
PE	11	4	15		10	3	13	
	5,151.01	832.070534	5,983.08	1.16	4,989.59	739.401411	5,729.00	1.15
NL	18	7	25		15	4	19	
	21,142.76	4,051.83	25,194.59	1.19	18,702.54	2,588.65	21,291.19	1.14
YT	16	11	27		11	8	19	
	1,029.355123	714.230286	1,743.59	1.69	817.24716	583.33571	1,400.58	1.71
NT	19	6	25		15	5	20	
	1,764.19	177.763272	1,941.95	1.10	1,694.94	136.400528	1,831.34	1.08
NU	15	14	29		9	8	17	
	1,251.19	1,594.08	2,845.27	2.27	1,036.35	511.617766	1,547.96	1.49
Total	317	255	572		240	144	384	
	988,114.51	855,500.56	1,843,615.07	1.87	702,464.68	661,095.32	1,363,560.0	1.94

**Table 7:** Age and gender breakdown for cases of AGI (*n* = 567) without adjustment for 225 missing responses and corresponding monthly prevalence of AGI based on weighted values.

	*n*	Weighted (*n*)	Weighted (%)	Monthly prevalence (AGI) with no adjustment for missing responses (%)
Gender				
Female	308	775,612	54	4.77
Male	259	650,748	46	3.86
Age group (years)				
0 to 9	215	267,052	19	7.27
10 to 19	131	207,560	15	5.01
20 to 64	139	855,838	60	4.19
65+	82	95,909	7	1.96

## Appendices

### A. Adjustment for Missing Data

#### A.1. Missing Data

Starting in November 2014 to the end of the survey time period (April 2015), due to an error in the CATI survey tool, 225 respondents (representing 30.5% of the respondents with any symptoms in the past 28 days) who indicated AGI symptoms were not asked all the relevant survey questions. These participants responded “yes” to having any symptoms of vomiting or diarrhea but “no” to vomiting symptoms specifically; therefore, it is assumed that they only had diarrhea symptoms. Questions missed pertained to the subsection specific to diarrhea and included duration of diarrhea symptoms, number of stools, and related chronic conditions or medication use. Information on respiratory symptoms was captured for all cases.

#### A.2. Approach to Adjustment

The responses from diarrhea-only cases from April to October (i.e., respondents where all questions were correctly asked) were adjusted to account for the diarrhea-only cases from November to April that had missing variables, so they could not be assessed if they met the case definition. Complete responses were given additional weight to account for those that were incomplete. The weighted value of the respondents with incomplete interviews (*n* = 855,500.56) was assigned to the weighted responses for the respondents with complete interviews (*n* = 988,114.51).

To estimate the more specific case definition of AGI without respiratory symptoms, a different adjustment was made as information on the respiratory symptoms was available for all respondents. The weighted value of the respondents with AGI and no respiratory symptoms but with incomplete interviews (*n* = 661,095.32) was assigned to the weighted responses for the respondents with AGI and no respiratory symptoms and complete interviews (*n* = 702,464.68). As this more specific definition necessitated cases not have concurrent respiratory symptoms and that information was available from all respondents, noncases could be identified directly, and thus, their true weight was incorporated into the noncase definition without need for adjustment.

Using this approach, province/territory-specific multipliers were developed for cases of AGI (regardless of respiratory symptoms) and for cases of AGI without respiratory symptoms (Table 6). Essentially, the weight of completed interviews from each province/territory was given additional weight to account for those with missing responses in that province. This approach assumed that the distribution of chronic disease, medical condition, or medication use as the cause of AGI symptoms would be the same over time and that the number of stools for cases occurring in April to October would have the same distribution as that for cases occurring in November to April. Similarly, care seeking behaviours, duration of illness, hospitalization, etc. would also have the same distribution.

##### A.3. Adjustment 1 Example for Cases of AGI (Regardless of Respiratory Symptoms)

(A.1) $$\begin{array}{c} \left(C\right)=\frac{\left(A+B\right)}{A}, \end{array}$$

where *A* = weighted value of complete interviews for diarrhea only; *B* = weighted value of incomplete interviews for diarrhea only; *C* = adjustment multiplier (province specific) for complete interviews for diarrhea only; *BC* multiplier: (172,666.37 + 85,951.20)/172,666.37 = 1.50.

Therefore, the individual complete diarrhea-only responses for *BC* are weighted an additional 1.5 times.

#### A.4. Adjustment 2 Example for Cases of AGI without Respiratory Symptoms

(A.2) $$\begin{array}{c} \left(Z\right)=\frac{\left(X+Y\right)}{X}, \end{array}$$

where *X* = weighted value of complete interviews for diarrhea only and no respiratory symptoms; *Y* = weighted value of incomplete interviews for diarrhea only and no respiratory symptoms; *Z* = adjustment multiplier (province/territory specific) for complete interviews for diarrhea only and no respiratory symptoms; *BC* multiplier: (159,460.83 + 44,916.74)/159,460.83 = 1.28.

Therefore, the individual complete diarrhea-only and no respiratory symptom responses for *BC* are weighted an additional 1.28 times.

#### A.5. Adjustment for Seasonality Comparison

To report on seasonality by symptom, a different approach was needed to account for the missing data. The proportion of cases that experienced diarrhea only from April to October was calculated (60.04% and 46.72% for the international AGI case definition and the more specific case definition where cases with concurrent respiratory symptoms were removed, resp.). This proportion was then multiplied by the weighted total of respondents reporting diarrhea only that had missing information for each month to generate the estimate of the monthly number of cases that would have experienced diarrhea only. This value was combined with the reported values for the other symptom profiles (i.e., vomiting only and both diarrhea and vomiting) to estimate the seasonality of each symptom profile by month.

(A.3) $$\begin{array}{c} H=\left(E\times \frac{F}{G}\right), \end{array}$$

where *E* = weighted value for month with incomplete interviews for diarrhea only; *F* = weighted value of completed interviews for diarrhea only that met case definition; *G* = weighted value of completed interviews for diarrhea only; *H* = estimated weighted value of respondents from month with incomplete data that met case definition.

#### A.6. Example

For January and the international AGI case definition, there were 47 respondents with a weighted value of 98,976, indicating that they experienced diarrhea symptoms, but with missing data, the weighted value of these respondents was multiplied by the proportion of completed weighted interviews that were diarrhea only (60.04% or 593,306.07/988,144.51) to estimate that 59,430 weighted respondents would have experienced diarrhea only in January. These values were then used to estimate the monthly prevalence by the symptom profile reported in Figure 2.

The difference of cases (98,967–59,430) would have been considered noncases that did not meet the case definition of having three or more loose stools in 24 hours or that their symptoms were related to a chronic condition or medication use.

### A.7. Limitations

As the survey was a weighted study design, it required a weighted analysis, and thus, it was necessary to devise a weighted adjustment to address the CATI survey error. Ideally, we would have liked to perform this adjustment based on province/territory + age + gender weights so that we could comment on the differences among these demographics. However, due to data limitations in the province/territory + age + gender combinations where certain combinations were missing, this was not possible. Of the interviews with missing data from November 2014 to April 2015, about 8% (19/225) of AGI cases and 12% (18/144) of AGI cases without respiratory symptoms did not have completed interview data, affecting 11 and 14 of the province/territory + age + gender combinations, respectively. Therefore, province/territory + age + gender combinations were not used in the adjustment, and results on demographics could not be reported. The differences between age + gender combinations are inherently captured in the province-specific adjustment but are not able to be described explicitly. Available age and gender data were explored, and AGI results indicated little demographic difference to previous NSAGI studies (e.g., higher rates in children and lower rates in 65 years+ age group).  Table 7 illustrates the demographic distribution of AGI cases with known responses and without adjustment for the missing 225 respondents for comparison with the adjusted values reported in the manuscript. This information however underestimates the true burden of AGI and thus cannot be used as the result for this research and is included only to demonstrate the general demographic conclusions that age and gender estimates do not differ greatly from what has been seen in previous NSAGI studies.

### B. Prevalence and Annual Incidence Rate Calculation

Formulas for calculating prevalence and annual incidence rate are as follows [31]:

(B.1) $$\begin{array}{c} \text{Prevalence}=\frac{\#\text{ofcases}}{\text{totalatrisk}}, \end{array}$$

(B.2) $$\begin{array}{c} \text{annualincidencerate}=\frac{\#\text{ofcases}}{1/2\left[\left(\text{total}\#\text{atrisk}\right)+\left(\text{total}\#\text{atrisk}\right)-\left(\#\text{ofcases}\right)\right]}\times \frac{365}{28}. \end{array}$$

## References

[1] Henson S. J., Majowicz S. E., Masakure O. (2008). Estimation of the costs of acute gastrointestinal illness in British Columbia, Canada. *International Journal of Food Microbiology*.

[2] Majowicz S. E., McNab W. B., Sockett P. (2006). Burden and cost of gastroenteritis in a Canadian community. *Journal of Food Protection*.

[3] Bartsch S. M., Lopman B. A., Ozawa S., Hall A. J., Lee B. Y. (2016). Global economic burden of norovirus gastroenteritis. *PLoS ONE*.

[4] World Health Organization (2015). *World Health Organization Foodborne Disease Burden Epidmiology Reference Group: World Health Organization Estimates of the Global Burden of Foodborne Diseases*.

[5] Jones T. F., McMillian M. B., Scallan E. (2007). A population-based estimate of the substantial burden of diarrhoeal disease in the United States; FoodNet, 1996-2003. *Epidemiology and Infection*.

[6] Thomas M. K., Perez E., Majowicz S. E. (2011). Burden of acute gastrointestinal illness in the Metropolitan region, Chile, 2008. *Epidemiology and Infection*.

[7] Flint J. A., van Duynhoven Y. T., Angulo F. J. (2005). Estimating the burden of acute gastroenteritis, foodborne disease, and pathogens commonly transmitted by food: an international review. *Clinical Infectious Diseases*.

[8] Imhoff B., Morse D., Shiferaw B. (2004). Burden of self-reported acute diarrheal illness in FoodNet surveillance areas, 1998-1999. *Clinical Infectious Diseases*.

[9] de Wit M. A., Koopmans M. P., Kortbeek L. M. (2001). Sensor, a population-based cohort study on gastroenteritis in the Netherlands: incidence and etiology. *American Journal of Epidemiology*.

[10] Scallan E., Fitzgerald M., Collins C. (2004). Acute gastroenteritis in Northern Ireland and the Republic of Ireland: a telephone survey. *Communicable Disease and Public Health*.

[11] Roberts J. A., Cumberland P., Sockett P. N. (2003). Infectious Intestinal Disease Study Executive: the study of infectious intestinal disease in England: socio-economic impact. *Epidemiology and Infection*.

[12] Wilking H., Spitznagel H., Werber D., Lange C., Jansen A., Stark K. (2013). Acute gastrointestinal illness in adults in Germany: a population-based telephone survey. *Epidemiology and Infection*.

[13] Adlam S. B., Perera S., Lake R. J., Campbell D. M., Williman J. A., Baker M. G. (2011). Acute gastrointestinal illness in New Zealand: a community study. *Epidemiology and Infection*.

[14] Scavia G., Baldinelli F., Busani L., Caprioli A. (2012). The burden of self-reported acute gastrointestinal illness in Italy: a retrospective survey, 2008-2009. *Epidemiology and Infection*.

[15] Aguiar Prieto P., Finley R. L., Muchaal P. K. (2009). Burden of self-reported acute gastrointestinal illness in Cuba. *Journal of Health, Population, and Nutrition*.

[16] Gurpreet K., Tee G. H., Amal N. M., Paramesarvathy R., Karuthan C. (2011). Incidence and determinants of acute diarrhoea in Malaysia: a population-based study. *Journal of Health, Population, and Nutrition*.

[17] Hall G. V., Kirk M. D., Ashbolt R., Stafford R., Lalor K. (2006). Frequency of infectious gastrointestinal illness in Australia, 2002: regional, seasonal and demographic variation. *Epidemiology and Infection*.

[18] Ho S. C., Chau P. H., Fung P. K., Sham A., Nelson E. A., Sung J. (2010). Acute gastroenteritis in Hong Kong: a population-based telephone survey. *Epidemiology and Infection*.

[19] Tam C. C., Rodrigues L. C., Viviani L. (2012). Longitudinal study of infectious intestinal disease in the UK (IID2 study): incidence in the community and presenting to general practice. *Gut*.

[20] Adak G. K., Long S. M., O’Brien S. J. (2002). Trends in indigenous foodborne disease and deaths, England and Wales: 1992 to 2000. *Gut*.

[21] Thomas M. K., Perez E., Majowicz S. E. (2010). Burden of acute gastrointestinal illness in Galvez, Argentina, 2007. *Journal of Health, Population, and Nutrition*.

[22] Thomas M. K., Majowicz S. E., MacDougall L. (2006). Population distribution and burden of acute gastrointestinal illness in British Columbia, Canada. *BMC Public Health*.

[23] Sargeant J. M., Majowicz S. E., Snelgrove J. (2008). The burden of acute gastrointestinal illness in Ontario, Canada, 2005-2006. *Epidemiology and Infection*.

[24] Majowicz S. E., Hall G., Scallan E. (2008). A common, symptom-based case definition for gastroenteritis. *Epidemiology and Infection*.

[25] Thomas M. K., Murray R., Flockhart L. (2013). Estimates of the burden of foodborne illness in Canada for 30 specified pathogens and unspecified agents, circa 2006. *Foodborne Pathogens and Disease*.

[26] Thomas M. K., Murray R., Flockhart L. (2015). Estimates of foodborne illness-related hospitalizations and deaths in Canada for 30 specified pathogens and unspecified agents. *Foodborne Pathogens and Disease*.

[27] Thomas M. K., Majowicz S. E., Sockett P. N. (2006). Estimated numbers of community cases of illness due to *Salmonella*, *Campylobacter* and verotoxigenic *Escherichia coli*: pathogen-specific community rates. *Canadian Journal of Infectious Diseases and Medical Microbiology*.

[28] Public Health Agency of Canada (2015). Infectious Disease Prevention and Control Branch.

[29] Canada. (code 01) and Canada (code 01) table (October 2012). Census profile. 2011 Census. Statistics Canada Catalogue no. 98-316-XWE. http://www12.statcan.gc.ca/census-recensement/2011/dp-pd/prof/index.cfm?Lang=E.

[30] Hall G., McDonald L., Majowicz S. E. (2010). Respiratory symptoms and the case definition of gastroenteritis: an international analysis of the potential impact on burden estimates. *Epidemiology and Infection*.

[31] Rothman K. J., Greenland S., Lash T. L. (2008). *Modern Epidemiology*.

[32] Wilson S. E., Rosella L. C., Wang J. (2016). Population-level impact of Ontario’s infant rotavirus immunization program: evidence of direct and indirect effects. *PLoS ONE*.

[33] Comeau J. L., Gagneur A., Quach C. (2016). Impact of a publicly funded monovalent rotavirus vaccination program in the province of Quebec (Canada). *Vaccine*.

[34] Scallan E., Hoekstra R. M., Angulo F. J. (2011). Foodborne illness acquired in the United States—major pathogens. *Emerging Infectious Diseases*.

[35] Muller L., Korsgaard H., Ethelberg S. (2012). Burden of acute gastrointestinal illness in Denmark 2009: a population-based telephone survey. *Epidemiology and Infection*.

[36] Majowicz S. E., Edge V. L., Fazil A. (2005). Estimating the under-reporting rate for infectious gastrointestinal illness in Ontario. *Canadian Journal of Public Health*.

[37] Harper S. L., Edge V. L., Ford J. (2015). Acute gastrointestinal illness in two Inuit communities: burden of illness in Rigolet and Iqaluit, Canada. *Epidemiology and Infection*.

[38] Febriani Y., Levallois P., Gingras S., Gosselin P., Majowicz S. E., Fleury M. D. (2010). The association between farming activities, precipitation, and the risk of acute gastrointestinal illness in rural municipalities of Quebec, Canada: a cross-sectional study. *BMC Public Health*.

[39] Hansdotter F. I., Magnusson M., Kuhlmann-Berenzon S. (2015). The incidence of acute gastrointestinal illness in Sweden. *Scandinavian Journal of Public Health*.

[40] Majowicz S. E., Dore K., Flint J. A. (2004). Magnitude and distribution of acute, self-reported gastrointestinal illness in a Canadian community. *Epidemiology and Infection*.

[41] Thomas M. K., Majowicz S. E., Pollari F., Sockett P. N. (2008). Burden of acute gastrointestinal illness in Canada, 1999-2007: interim summary of NSAGI activities. *Canada Communicable Disease Report*.

[42] Edge V. L., Odoi A., Fyfe M. (2007). Physician diagnostic and reporting practices for gastrointestinal illnesses in three health regions of British Columbia. *Canadian Journal of Public Health*.

[43] Tam C. C., Rodrigues L. C., O’Brien S. J. (2003). The study of infectious intestinal disease in England: what risk factors for presentation to general practice tell us about potential for selection bias in case-control studies of reported cases of diarrhoea. *International Journal of Epidemiology*.

[44] Viviani L., van der Es M., Irvine L. (2016). Estimating the incidence of acute infectious intestinal disease in the community in the UK: a retrospective telephone survey. *PLoS ONE*.

[45] Cantwell L. B., Henao O. L., Hoekstra R. M., Scallan E. (2010). The effect of different recall periods on estimates of acute gastroenteritis in the United States, FoodNet Population Survey 2006-2007. *Foodborne Pathogens and Disease*.

